# Recognizing Declining Right Ventricular Reserve in Pediatric Pulmonary Arterial Hypertension: A Clinical Framework for Discordant Multimodal Findings

**DOI:** 10.3390/jcm15218215

**Published:** 2026-10-22

**Authors:** Xiaolan Zheng, Yimin Hua, Kaiyu Zhou

**Affiliations:** Key Laboratory of Birth Defects and Related Diseases of Women and Children of MOE, Department of Pediatrics, West China Second University Hospital, Sichuan University, Chengdu 610041, China

**Keywords:** pediatric pulmonary arterial hypertension, right ventricular reserve, right ventricular decompensation, ventricular-arterial coupling, cardiovascular magnetic resonance, right-heart catheterization

## Abstract

Right ventricular (RV) failure is a major determinant of clinical deterioration in pediatric pulmonary arterial hypertension (PAH), yet loss of RV reserve is difficult to recognize before overt low-output failure. Pulmonary artery pressure may remain stable or fall as RV pressure-generating capacity declines. Other commonly followed variables may also diverge: tricuspid annular plane systolic excursion can remain preserved despite worsening RV free-wall strain or cardiovascular magnetic resonance (CMR)-derived ejection fraction; natriuretic peptides may rise before conventional echocardiographic changes; and symptoms may deteriorate while estimated pressure appears unchanged. This focused narrative review integrates pediatric evidence with selected physiologic data to examine the interpretation of such discordance. We distinguish three clinical states—maintained adaptation, suspected loss of reserve, and overt decompensation—without proposing a new risk score. Assessment is organized around the clinical question left unanswered after the initial review. Signal quality and serial comparability are checked first, followed by transient modifiers of loading conditions and biomarker concentration. CMR helps resolve uncertainty about RV volume or global function, whereas right-heart catheterization directly defines pulmonary vascular load, filling pressure, and forward output when these measurements will affect management. Direct pediatric evidence supports serial assessment of RV and right atrial remodeling, myocardial deformation, CMR-derived function, natriuretic peptide trajectories, right atrial pressure, cardiac index, and mixed venous oxygen saturation, but no isolated variable has been validated as an independent treatment threshold. Concordance, direction, and rate of change across domains may improve recognition of declining RV reserve while limiting escalation driven by measurement noise.

## 1. Introduction

A fall in estimated pulmonary artery pressure is usually reassuring only when forward flow and right-heart function are preserved. In pediatric PAH, the same observation can also mark a failing ventricle that can no longer generate its previous pressure. A second common conflict is a preserved tricuspid annular plane systolic excursion (TAPSE) despite progressive RV dilatation, impaired free-wall strain, or a lower CMR-derived RV ejection fraction (RVEF). A third is a sustained rise in B-type natriuretic peptide (BNP) or N-terminal pro-BNP (NT-proBNP) without a clear change on conventional echocardiography. Such discrepancies can obscure early loss of RV reserve, whereas measurement noise can prompt unnecessary escalation.

Contemporary PAH guidance emphasizes repeated, multidimensional assessment rather than reliance on pulmonary artery pressure alone [1,2,3]. Miles et al. recently reviewed echocardiography and CMR across the major components of RV physiology in pediatric pulmonary hypertension [4]. The present review addresses a follow-up problem within that broader assessment: what to do when serial measurements disagree. We examine how the credibility of an adverse change depends on acquisition conditions, its persistence, and accompanying findings, and how the remaining uncertainty determines the next investigation. The proposed framework complements established risk assessment by helping clinicians interpret conflicting inputs before assigning them clinical weight.

This review focuses on World Health Organization group 1 pediatric PAH from infancy through adolescence. Broader pediatric pulmonary hypertension cohorts are included when they inform RV physiology or measurement interpretation relevant to PAH. Age, body size, developmental capacity to report symptoms, shunt physiology, and sedation or anesthesia are considered when applying these findings. Adult evidence supplies physiologic context and is distinguished from direct pediatric observations. Declining RV reserve refers here to a reproducible adverse trajectory suggesting narrowing adaptive capacity before established low output; overt RV decompensation denotes clinical or hemodynamic evidence that effective forward flow can no longer be maintained.

## 2. Literature Search and Evidence Scope

A focused PubMed/MEDLINE search was performed through 20 August 2026 using combinations of the terms pediatric, child, pulmonary arterial hypertension, pulmonary hypertension, right ventricle, right atrium, remodeling, decompensation, strain, echocardiography, cardiovascular magnetic resonance, ventricular–arterial coupling, hemodynamics, cardiac index, mixed venous oxygen saturation, BNP, and NT-proBNP. Reference lists of pediatric guidelines, World Symposium reports, recent imaging reviews, and eligible original studies were screened for additional publications. The publications cited in this review were in English. Study identification and primary selection were performed by X.Z. Priority was given to original pediatric cohorts with longitudinal, prognostic, hemodynamic, reproducibility, or clinically interpretable imaging data. Mixed pediatric pulmonary hypertension cohorts were retained when the population was described and the right-heart findings were relevant to PAH. Adult studies were used for foundational physiology or measurement interpretation when pediatric data were insufficient.

Selected pediatric studies inform interpretation of RV and right atrial (RA) findings, distinguishing longitudinal prognostic evidence from hemodynamic associations and registry characterization. The clinical interpretations developed in the text are not derived or validated prediction models. The review is narrative, and no pooled estimate was attempted because populations, acquisition methods, and outcomes differ. Physiologic explanations and the proposed assessment pathway are distinguished from findings directly tested in pediatric cohorts.

## 3. Right Ventricular Reserve as a Load–Contractility Relationship

### 3.1. Afterload Extends Beyond Pulmonary Vascular Resistance

The RV is adapted to a low-pressure, highly compliant circulation. Physiologic studies, drawn largely from adult and experimental work, show that afterload comprises pulmonary vascular resistance (PVR), pulmonary arterial compliance, characteristic impedance, wave reflection, and pulsatile load [5,6]. Applied to children, this explains why the same indexed PVR need not impose the same effective load, although it does not establish pediatric thresholds. Pulmonary artery pressure also depends on flow and the ventricle’s ability to generate pressure. It therefore cannot describe vascular disease severity or RV performance on its own.

### 3.2. RV–Pulmonary Arterial Coupling: Physiologic Context and Current Limitations

RV-pulmonary arterial coupling describes whether contractility is sufficient for the prevailing afterload. The pressure-volume formulation using end-systolic and arterial elastance provides the physiologic basis [5,6]. Pediatric investigations have assessed more accessible surrogates, including the stroke volume/end-systolic volume ratio and combinations of echocardiographic and hemodynamic measurements [7,8]. In 108 children with PAH studied at two centers, Simpkin et al. found that selected strain/load ratios predicted death or transplantation, whereas free-wall strain and TAPSE alone did not; the added prognostic information over load variables was limited [9]. This finding supports interpretation of myocardial deformation alongside afterload rather than an assumption that strain is invariably the superior marker. These ratios remain surrogates, and their performance depends on the measures and population used.

### 3.3. Loss of Reserve Precedes Overt Failure

**Figure 1:**
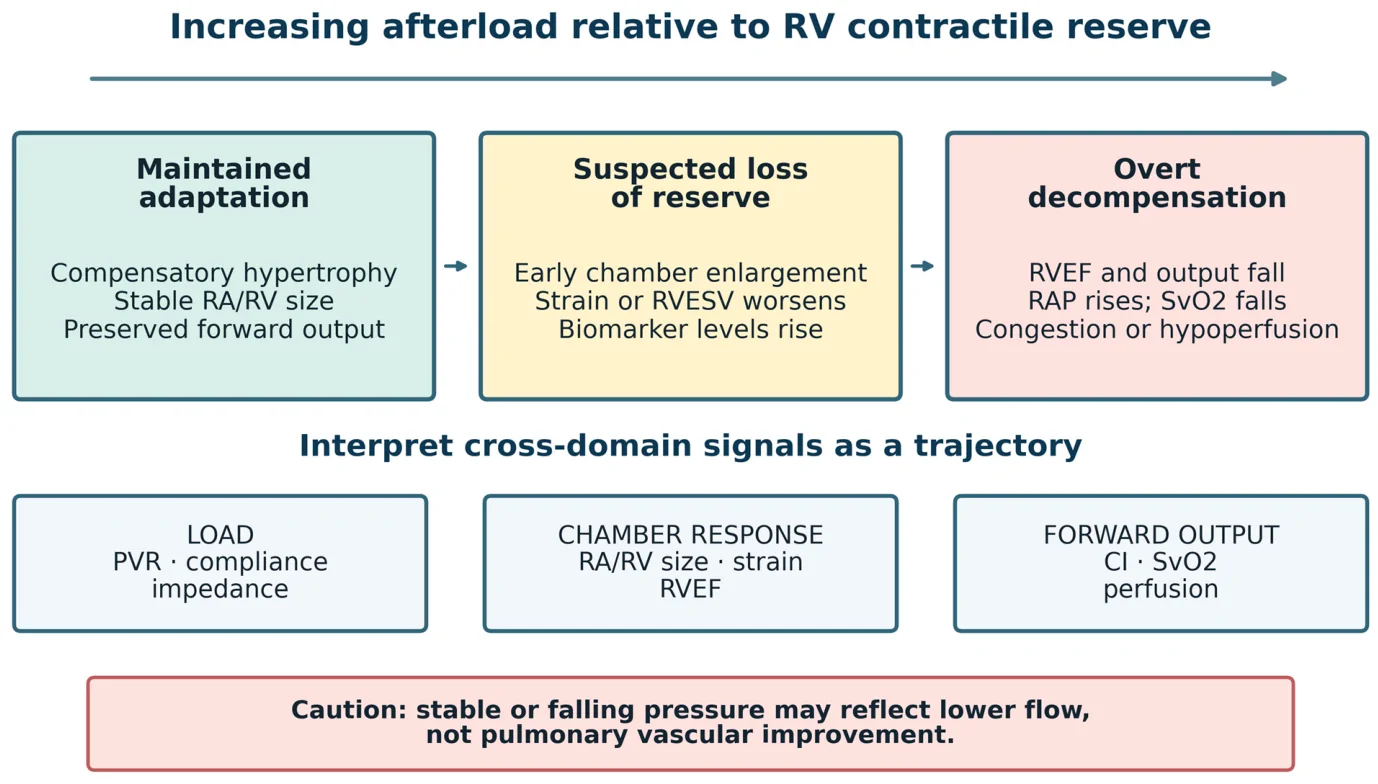
Conceptual continuum from maintained RV adaptation to declining reserve and overt decompensation in pediatric PAH. Abbreviations: CI, cardiac index; PVR, pulmonary vascular resistance; RA, right atrium; RAP, right atrial pressure; RV, right ventricle; RVEF, right ventricular ejection fraction; RVESV, right ventricular end-systolic volume; SvO2, mixed venous oxygen saturation. This is a clinical integration framework, not a validated staging system.

Adaptation initially involves increased contractility and hypertrophy sufficient to maintain stroke volume. The progression from adaptation to failure is a physiologic model informed largely by adult and experimental evidence [5,6]. Pediatric studies support associations between adverse outcomes and RV deformation, chamber remodeling, RA function, and biomarkers, but have not established a uniform sequence in which these abnormalities appear. Resting imaging and biomarker trajectories are therefore indirect evidence of narrowing reserve, rather than direct measurements of the ability to augment output during stress. Overt decompensation includes impaired perfusion or congestion with evidence that the RV can no longer sustain adequate forward flow (Figure 1).

RA findings add information about how the right heart accommodates that load. In a multicenter CMR cohort of 72 children with PAH, reduced conduit function coexisted with greater active atrial contribution; conduit function and the active-to-conduit ratio were associated with subsequent adverse events during a median 3.2-year follow-up [10]. Greater atrial contraction can thus accompany compensatory dependence on the atrium, rather than signify recovery. Pediatric echocardiographic studies also link RA deformation and phasic function to outcomes [11,12,13]. Interpretation should consider which phase is changing and its relation to RV size and filling pressure.

## 4. Why Multimodal Findings Diverge in Children

### 4.1. Variables Interrogate Different Links in the Decompensation Pathway

TAPSE and tissue Doppler S’ emphasize basal longitudinal motion; fractional area change depends on endocardial definition in a complex chamber; strain measures myocardial deformation; CMR quantifies global volumes, mass, and ejection fraction; natriuretic peptides reflect myocardial wall stress; and invasive hemodynamics describe load, filling pressure, and effective forward output. These measurements may diverge because they sample different aspects of the disease. Multimodal discordance is defined here as a persistent or reproducible divergence in the direction or extent of change across at least two measurements, modalities, or clinical domains after technical and physiologic explanations have been considered. This includes disagreement between imaging measures as well as between imaging, symptoms, biomarkers, and hemodynamics. A universal numeric change in TAPSE, strain, or RVEF is not specified because pediatric PAH-specific minimal detectable and clinically important changes have not been established across ages and platforms.

### 4.2. Development and Phenotype Modify Interpretation

RV dimensions, heart rate, longitudinal excursion, and loading conditions change across childhood. A fixed adult threshold can misclassify both small children and adolescents. Etiology also matters. Idiopathic or heritable PAH, repaired congenital heart disease-associated PAH, persistent shunts, and developmental lung disease expose the RV to different combinations of pressure, volume, hypoxemia, and ventricular interaction [2,3,14,15,16]. Interpretation of the right-heart phenotype therefore requires definition of anatomy, shunt direction, repair status, lung disease, and oxygenation.

### 4.3. Acquisition Conditions Can Create False Discordance

Before discordance is attributed to disease progression, serial studies should be checked for comparable image quality, RV-focused views, tracing conventions, vendor and software version, heart rate, rhythm, preload, oxygen, ventilation, vasoactive medication, fever, anemia, and sedation or anesthesia. Aly et al. studied 70 infants and children with normal cardiac anatomy and varying ventricular function [17]. RV strain agreement between analysis vendors was lower than within-vendor reproducibility, particularly with compressed data or unfavorable frame-rate/heart-rate relationships. An apparent change after a platform switch should therefore prompt reanalysis in a consistent environment where feasible.

For CMR, van der Ven et al. analyzed a healthy pediatric reference population and found greater observer reproducibility for ventricular volumes than for ejection fraction [18]. This study assessed repeat analysis of images; it does not quantify all sources of variability between separate scans in children with PAH. Contouring, acquisition conditions, interval growth, and loading remain relevant. Neither study establishes a PAH-specific minimal detectable change. Catheter measurements require additional review of zeroing, wedge-pressure credibility, oxygen-consumption assumptions, and flow estimation in the presence of shunts [19,20,21]. Differences acquired under substantially different conditions cannot be assumed to represent disease progression.

## 5. Clinically Important Discordance Patterns

### 5.1. Estimated Pulmonary Artery Pressure Falls While the RV Enlarges or Output Worsens

A lower Doppler-estimated pressure can accompany improvement in vascular load, reduced flow, or loss of RV pressure-generating capacity. It becomes concerning when RA/RV enlargement, worsening function, increasing tricuspid regurgitation (TR), rising BNP/NT-proBNP, syncope, or impaired perfusion develops at the same time. The TR envelope, estimated RA pressure, systemic blood pressure, and shunt direction should first be reviewed. A weaker or incomplete TR signal can underestimate pressure, while changing shunt flow can alter both pressure and chamber volume [22,23].

The useful distinction is whether lower pressure accompanies preserved or improving forward flow. Stable activity, less congestion, and improving RV size and function support a favorable response. Increasing RV end-systolic volume, lower measured output, or worsening perfusion support concern about ventricular failure. CMR can establish whether global remodeling and function have changed; right-heart catheterization (RHC) is needed when uncertainty about load, filling pressure, or output will affect treatment. These are physiologically reasoned interpretations within multidimensional pediatric assessment [1,2,3,19,20,21], rather than a pediatric diagnostic rule validated specifically for falling pressure.

### 5.2. Preserved TAPSE with Worsening Strain or CMR-Derived Function

TAPSE is accessible and reproducible but samples basal longitudinal motion and is influenced by translation and loading. In Okumura et al.’s retrospective cohort of 17 children with idiopathic PAH, serial strain deteriorated among the six children who died or underwent transplantation, while remaining stable in survivors [24]. This supports attention to a reproducible deformation trajectory, although the small cohort does not define a portable change threshold. Conventional measures also retain value: in 63 children with PAH followed for a median four years, Lammers et al. linked RA area and left ventricular (LV) diastolic eccentricity to transplant-free survival, with TAPSE contributing to mortality prediction [25]. This study supports integrated conventional echocardiography rather than a strain-specific claim.

CMR adds a different view of the ventricle. Ta et al. studied 89 children with PH, 84% with group 1 disease; 21 experienced a composite of death, lung transplantation, Potts shunt, or parenteral prostacyclin initiation [26]. Larger RV volumes and lower RVEF and global function index were associated with events. These findings support assessment of global remodeling when annular excursion appears reassuring. However, strain remains sensitive to software, frame rate, image quality, and load [17], while CMR depends on contouring and acquisition conditions [18]. Neither modality automatically overrides the other.

Preserved TAPSE with less negative free-wall strain is more concerning when RV end-systolic volume or RA size also increases, activity declines, or biomarkers rise. An isolated strain difference after a software change has a different evidentiary weight. CMR is useful when the unresolved question is whether the whole RV has enlarged or its global function has declined. If the uncertainty concerns whether output is inadequate for the prevailing load, the question is hemodynamic and may require RHC. This choice follows the suspected mechanism, rather than a fixed hierarchy of imaging tests.

### 5.3. Natriuretic Peptides Rise While Conventional Echocardiography Appears Stable

Said et al. followed 82 children with PAH, measuring NT-proBNP and other severity markers every three to six months over a median 4.8 years [27]. Serial concentrations were associated with disease severity and transplant-free survival, and trajectories differed between survivors and nonsurvivors. This provides direct pediatric support for repeated measurements. It does not establish that NT-proBNP consistently rises before imaging changes, or that a biomarker-guided treatment strategy improves outcomes.

A sustained rise should be interpreted against renal function, infection, hypoxemia, anemia, volume status, and the assay used. Persistence after a transient modifier resolves, or concurrence with reduced activity or subtle remodeling, increases concern. A normal-looking TAPSE alone is insufficient to dismiss that pattern. Earlier reassessment is appropriate, with CMR considered when RV volume or global function remains uncertain. The urgency of invasive assessment depends on associated clinical deterioration and the hemodynamic question, rather than the biomarker concentration alone.

### 5.4. Symptoms or Daily Activity Worsen While Estimated Pressure Is Stable

Clinical deterioration takes priority over an isolated stable pressure estimate. In infants and young children, reduced intake, longer feeding time, diaphoresis, poor weight gain, less play, or cyanosis with crying may replace a conventional report of exercise intolerance. In older children, syncope, reduced school or recreational activity, and repeated hospitalization are important signals. Objective activity monitoring can support the history, although it remains an adjunct [28,29]. Arrhythmia, anemia, infection, lung disease, medication effects, and deconditioning should be considered alongside PAH progression.

The child’s previous activity provides a useful reference, provided circumstances are comparable. A reproducible decline in ordinary play or walking carries more weight than a change explained by an intercurrent illness or altered opportunity for activity. Persistent unexplained limitation should prompt assessment for low output even when estimated pressure is unchanged. Syncope, congestion, or impaired perfusion increase urgency; a scheduled repeat scan should not delay specialist evaluation [2,3,15].

### 5.5. A Single Imaging Variable Worsens While the Child, Biomarkers, and Other Domains Remain Stable

A mild isolated imaging change warrants review of quality and acquisition conditions before it is labeled deterioration. This applies to strain, derived coupling ratios, and CMR indices as well as conventional dimensions. Repeat analysis of the same images can identify reader or contouring differences, but a repeat acquisition may be required to test whether the finding persists. For a clinically stable child, the timing of reassessment should reflect the size and speed of the apparent change, baseline risk, and confidence in the measurement.

Corroboration should also be judged by how independent the observations are. RVEF and RV end-systolic volume share contour-derived information, and a ratio can change because its numerator or denominator changes. Agreement among such measures is less persuasive than a reproducible imaging change accompanied by altered activity, biomarker trajectory, or measured output. Existing pediatric prognostic associations support this integrated interpretation [4,7,8,9,24,25,26], but they do not determine a universal number of abnormal variables needed to diagnose loss of reserve.

### 5.6. What the Pediatric Evidence Supports

The studies in Table 1 answer different questions. Serial strain and NT-proBNP cohorts support longitudinal associations [24,27]; conventional echo and CMR cohorts associate measurements with later outcomes [25,26]; coupling studies evaluate combined function/load measures [7,8,9]. A biomarker or imaging association is not evidence that the full pattern of discordance predicts deterioration. Moreover, composites that include treatment escalation or hospitalization partly reflect clinical decisions and should not be treated as equivalent to death or transplantation. The present framework uses these observations to organize reassessment; whether it adds information beyond current risk assessment remains a question for prospective testing.

## 6. Question-Directed Escalation of Assessment

### 6.1. Standardized Echocardiography Is the Longitudinal Anchor

Echocardiography remains the practical basis of serial pediatric assessment. Its value increases when a stable acquisition set is used: RV-focused apical imaging; RA and RV size; septal configuration; tricuspid regurgitation severity; TAPSE; fractional area change; tissue Doppler S’; free-wall strain when technically adequate; and pericardial effusion [22,23]. Reports need to preserve raw values, body-size correction where appropriate, image quality, heart rate, rhythm, oxygen, ventilation, and sedation status. Repeating a questionable study under comparable conditions is often more informative than adding several nonstandard measurements.

### 6.2. Use CMR to Define Global Remodeling and Function

CMR is most useful when echocardiographic windows are inadequate; symptoms, biomarkers, and echocardiography diverge; global RV volume and ejection fraction are needed; anatomy is complex; or a major therapeutic decision requires a more reproducible baseline [30,31]. It can quantify RV end-diastolic and end-systolic volumes, RVEF, mass, pulmonary artery flow, and shunt volume without geometric assumptions. Emerging CMR-derived integrated indices, right atrial functional measures, and machine-learning approaches are promising [10,26,32], but they remain adjuncts to invasive hemodynamics. In young children, the potential value of CMR must be balanced against sedation or anesthesia risk and the possibility that physiologic conditions will differ from routine follow-up.

### 6.3. Use Right-Heart Catheterization When Load, Filling Pressure, or Output Must Be Defined

Right-heart catheterization is warranted when the answer is likely to change management and cannot be obtained noninvasively. Relevant scenarios include confirmation and classification at diagnosis; unexplained clinical deterioration; discordant noninvasive findings with concern for low output; inadequate response to therapy; assessment before major escalation or advanced intervention; and evaluation of congenital shunt operability [14,15,19,20,21]. Mean pulmonary artery pressure and indexed PVR require interpretation alongside right atrial pressure, cardiac index, mixed venous oxygen saturation, wedge pressure, oxygenation, flow method, and shunt state. Acute vasoreactivity testing answers a specific pharmacologic question and does not measure long-term RV reserve; echocardiographic prediction of its invasive response remains investigational [33].

### 6.4. Clinical Findings and Biomarkers Establish Urgency

**Figure 2:**
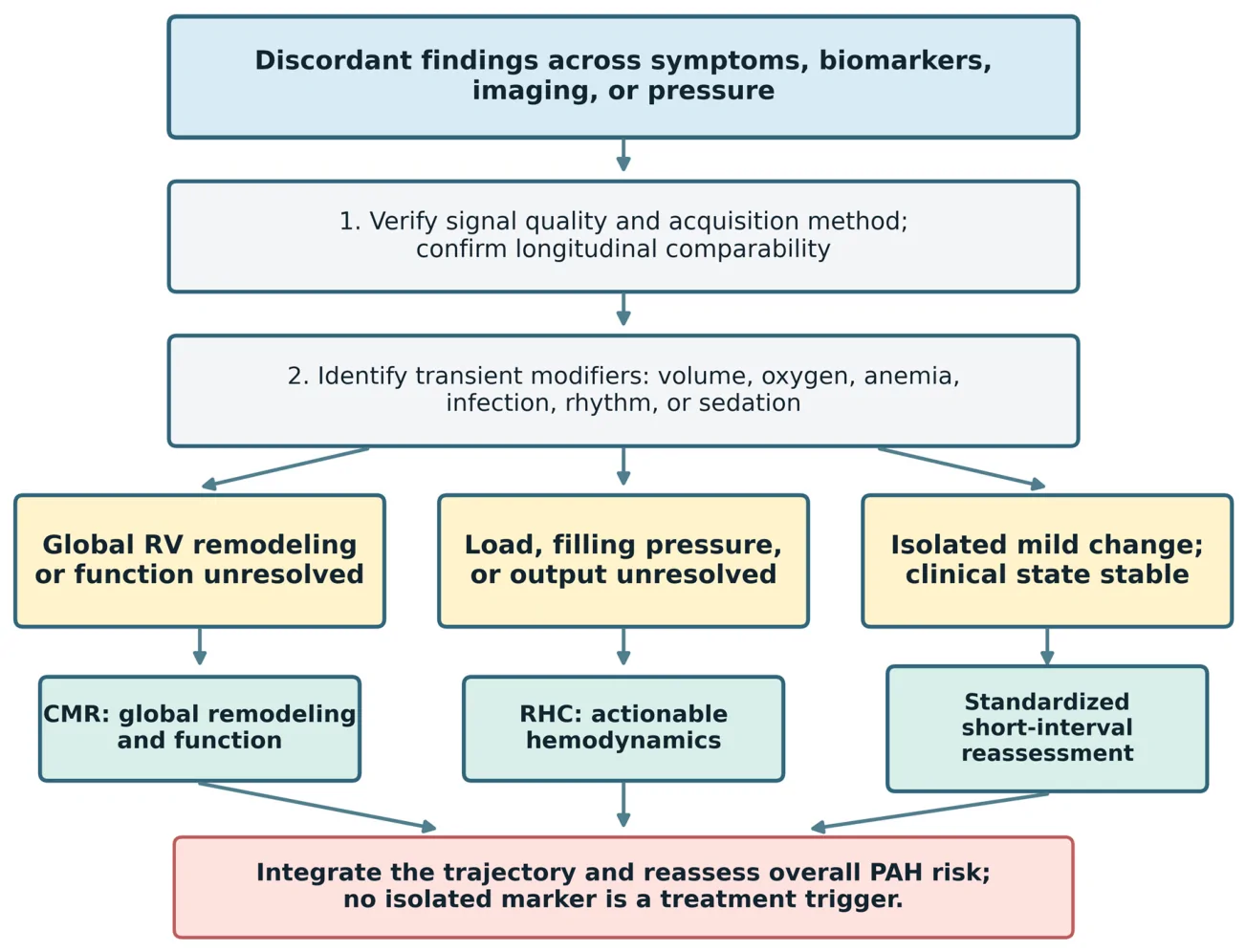
Clinical pathway for discordant right-heart findings in pediatric PAH. The pathway selects the next test according to the unresolved clinical question. CMR and RHC are complementary rather than sequential requirements. Abbreviations: CMR, cardiovascular magnetic resonance; PAH, pulmonary arterial hypertension; RHC, right-heart catheterization; RV, right ventricle.

BNP/NT-proBNP, symptoms, physical examination, growth, and age-appropriate activity provide repeated information at low procedural burden. Within this approach, they indicate whether an imaging abnormality is corroborated and whether the reassessment interval needs to be shortened. Syncope, overt right-heart failure, low blood pressure, worsening cyanosis, reduced urine output, cool extremities, progressive hepatomegaly, or recurrent hospitalization warrant prompt specialist reassessment even if a recent pressure estimate was stable. Table 2 summarizes the main discordance patterns, and Figure 2 outlines the question-directed assessment pathway.

## 7. An Integrated Clinical Framework

For practical communication, right-heart status can be described using three clinical states (Table 3). These are author-proposed heuristics, have not been prospectively evaluated, and are not validated risk categories or treatment thresholds. Maintained adaptation denotes stable activity, chamber size, function, and biomarkers with preserved output for the prevailing load. Suspected loss of reserve denotes a reproducible adverse trajectory with corroboration, or persistent clinically important discordance, before established low output. Overt decompensation denotes clinical or hemodynamic evidence of impaired forward flow or excessive right-sided filling pressure. Classification requires interpretation of the whole pattern, including etiology, anatomy, current therapy, and adherence [34,35,36].

The practical aim is to identify what remains uncertain. A child may need confirmation of an imaging change, a better estimate of global function, or direct measurement of output and load. These questions call for different investigations, and the urgency depends on clinical status. CMR and RHC may be complementary; neither is a mandatory intermediate step before every management decision.

### Illustrative Clinical Interpretation

The following hypothetical examples illustrate the reasoning and are not observations from a patient cohort. A clinically stable child has unchanged age-adjusted TAPSE but less negative free-wall strain after a change in analysis software. Activity, NT-proBNP, RA size, and RV dimensions are stable. The immediate question is whether the strain difference is reproducible. Reanalysis with the previous method, if available, or a standardized repeat study is more informative than assigning a new clinical state from the isolated value.

In a second example, TAPSE is similarly preserved, but less negative strain is reproduced on comparable studies and is accompanied by increasing RV end-systolic volume, rising NT-proBNP, and reduced ordinary activity. The concurrence makes a technical explanation less satisfactory. CMR can clarify global remodeling if this remains uncertain; RHC becomes relevant when forward output, filling pressure, or pulmonary vascular load must be defined to guide treatment. New syncope, congestion, or impaired perfusion would warrant urgent assessment. The distinction between the examples lies in the corroborating trajectory and clinical question, rather than the strain change alone.

## 8. Limitations

Large longitudinal pediatric cohorts remain scarce. Available studies differ in PAH subtype, developmental stage, treatment era, imaging platform, and outcome definition, and many are retrospective. Mixed-etiology PH cohorts and healthy reference populations supply useful observations but cannot establish PAH-specific longitudinal thresholds. Several prognostic composites include clinician-dependent events, and referral cohorts may not represent younger children or patients with different shunt anatomy. Reserve itself is generally inferred from resting measurements rather than directly measured during stress. The selective narrative search may also have missed relevant reports. Most importantly, no study has tested the proposed discordance pathway against usual multidimensional assessment or shown that acting on it improves outcomes.

## 9. Future Directions

Multicenter prospective pediatric PAH registries should collect serial symptoms and activity, biomarkers, standardized echocardiography, CMR when feasible, and clinically indicated invasive hemodynamics. Paired observations are needed to determine whether a discordant finding precedes another abnormality, remains isolated, or resolves when loading or acquisition conditions change. Strain studies should establish age- and vendor-specific measurement variability using agreed acquisition and post-processing standards, with separate assessment of repeat analysis and repeat acquisition. Analyses should account for PAH phenotype, shunt status, and developmental stage.

AI-enabled imaging biomarkers warrant evaluation within these datasets, including external validation across centers, equipment, and age groups. The relevant test is whether they add reliable information beyond standardized imaging, biomarkers, and established risk assessment, not simply whether they correlate with catheter measurements [32].

Prediction and clinical benefit require separate evaluation. Prospective studies should compare prespecified discordance-triggered assessment with usual care, measuring detection of clinically important low-output physiology, the number and consequences of additional investigations, and the timing of treatment decisions. A pathway that detects more abnormalities but causes unnecessary invasive procedures would have limited value. Demonstrating earlier recognition of deterioration with an acceptable investigation burden would provide a stronger basis for clinical use.

## 10. Conclusions

Discordant findings in pediatric PAH become informative when their technical credibility and clinical context are established. A reproducible adverse trajectory can justify closer assessment even when pulmonary artery pressure or TAPSE appears reassuring. The unresolved question should determine whether repeat echocardiography, CMR, or invasive hemodynamics is needed. The proposed framework organizes that reasoning; prospective comparison with existing risk assessment is required before it can be adopted as a validated clinical pathway.

## Figures and Tables

**Table 1:** Selected pediatric evidence informing assessment of RV reserve and decompensation.

Domain/Study	Population and Follow-Up	Main Measure	Outcome or Association	Interpretive Boundary	Evidence Type
RV deformation/Okumura et al. [24]	17 children with idiopathic PAH; echo at 3–6 months, then yearly	RV longitudinal strain	Six deaths/transplants; strain worsened in those with events	Small retrospective cohort; no validated change threshold	Longitudinal prognostic association
RV-PA coupling/Jone et al. [7]	125 children with PH; 65 controls; studied 2014–2017	3D echo SV/ESV	Associated with adverse clinical events, including escalation and hospitalization	Volume surrogate; composite includes clinician-dependent events	Prognostic association
Function/load ratios/Simpkin et al. [9]	108 children with PAH at two centers; simultaneous echo and catheterization	Free-wall strain or TAPSE combined with load	Selected ratios predicted death/transplant; strain and TAPSE alone did not	Limited incremental information over load; phenotype matters	Hemodynamic and prognostic association
Conventional echo/Lammers et al. [25]	63 children with PAH; no relevant shunts; median follow-up 4.0 years	RA area, LV eccentricity, TAPSE	17 deaths and four lung transplants; RA area and eccentricity predicted transplant-free survival	Observational referral cohort; score requires external validation	Prognostic association
CMR/Ta et al. [26]	89 children with PH; 84% group 1	RV volumes, RVEF, global function index	21 composite events: death, lung transplant, Potts shunt, or parenteral prostacyclin initiation	Mixed etiologies; composite includes treatment initiation; GFI uses standard CMR data	Prognostic association
RA CMR/Miles et al. [10]	72 children with PAH; 36 controls; median follow-up 3.2 years	RA volumes and phasic function	Reduced conduit function and higher active/conduit ratio associated with severity and adverse events	Retrospective; bivariate adjustment; active contribution may be compensatory	Phenotype and prognostic association
RA echo/Jone et al. [11]; Frank et al. [12]; Kumar et al. [13]	Pediatric PH/PAH cohorts; methods and follow-up differ	RA deformation, conduit fraction, active emptying	Associations with clinical worsening, adverse events, or survival	Phasic measures differ; greater active emptying need not indicate recovery	Longitudinal/prognostic association
NT-proBNP/Said et al. [27]	82 children with PAH; measurements every 3–6 months; median follow-up 4.8 years	Serial NT-proBNP	Trajectory associated with disease severity and death/transplant	No proof of a uniform lead time over imaging or benefit of biomarker-guided treatment	Longitudinal prognostic association
Hemodynamics/Beghetti et al. [19]	472 children with PH; 908 catheterizations in TOPP registry	Hemodynamics and procedural context	Describes diagnostic profiles and catheterization complications	Registry characterization; not a test of discordance or treatment thresholds	Registry characterization

Abbreviations: CMR, cardiovascular magnetic resonance; ESV, end-systolic volume; GFI, global function index; LV, left ventricle; PAH, pulmonary arterial hypertension; PH, pulmonary hypertension; RA, right atrium; RV, right ventricle; RV-PA, right ventricular-pulmonary arterial; RVEF, RV ejection fraction; SV, stroke volume. Selected studies are summarized according to their principal contribution; evidence types are descriptive, not formal certainty grades.

**Table 2:** Common discordance patterns and a question-directed response.

Discordant Pattern	Priority Interpretation	First Verification	Question-Directed Next Step
Estimated PA pressure falls, but RV/RA enlarge or function worsens	Falling RV pressure-generating capacity or lower flow; not necessarily pulmonary vascular improvement	TR envelope, shunt direction, volume status, oxygen/ventilation	CMR for global remodeling; RHC if output, RAP, or PVR will change management
TAPSE preserved, but strain or CMR-derived RVEF worsens	Regional motion may remain preserved while global reserve declines	RV-focused view, vendor/software, loading, serial comparability	Integrate RVESV, RA size/function, biomarkers, and activity; repeat imaging or use CMR to define global change
BNP/NT-proBNP rises, conventional echo appears stable	Early wall stress or noncardiac confounding	Renal function, infection, anemia, hypoxemia, volume, assay	Shorten interval; consider CMR if sustained and clinically concordant
Symptoms/activity worsen, estimated pressure stable	Low output, arrhythmia, pulmonary/comorbid disease, or measurement error	Clinical history, rhythm, hemoglobin, oxygenation, adherence	RHC when persistent decline is unexplained and actionable
Single mild echo abnormality, other domains stable	Measurement variability or transient load change	Same reader/team and acquisition conditions	Standardized short-interval repeat; do not escalate therapy on one noisy signal
CMR volume or RVEF worsens, child and biomarker stable	Early remodeling, measurement difference, or preclinical reserve loss	Sequence, contouring, physiologic conditions, interval growth	Confirm trajectory and integrate strain/output; avoid single-scan treatment decisions

Abbreviations: BNP, B-type natriuretic peptide; CMR, cardiovascular magnetic resonance; NT-proBNP, N-terminal pro-B-type natriuretic peptide; PA, pulmonary artery; PVR, pulmonary vascular resistance; RA, right atrium; RAP, right atrial pressure; RHC, right-heart catheterization; RV, right ventricle; RVEF, right ventricular ejection fraction; RVESV, right ventricular end-systolic volume; TAPSE, tricuspid annular plane systolic excursion; TR, tricuspid regurgitation.

**Table 3:** Clinical states of RV adaptation and their assessment implications.

Clinical State	Concordant Pattern	Interpretation	Assessment Implication
Maintained adaptation	Stable symptoms/activity; no progressive RA/RV enlargement; stable function and biomarker trajectory; preserved output	RV remains adequate for the prevailing load	Continue standardized longitudinal surveillance; compare with the child’s own baseline
Suspected loss of reserve	Reproducible worsening in at least one RV/RA domain with corroboration or a persistent clinically important discordance	Early adverse remodeling may precede resting low output	Verify acquisition and modifiers; shorten interval; select CMR or RHC according to the unresolved question
Overt decompensation	Syncope, right-heart failure, low perfusion, marked functional decline, rising RAP, falling CI or SvO2	RV no longer maintains effective forward flow	Urgent specialist reassessment and comprehensive risk/treatment review; do not await a higher PA pressure

Abbreviations: CI, cardiac index; PA, pulmonary artery; RA, right atrium; RAP, right atrial pressure; RHC, right-heart catheterization; RV, right ventricle; SvO2, mixed venous oxygen saturation. The three states are unvalidated descriptive heuristics, not a score or treatment algorithm.

## Data Availability

No new data were created or analyzed in this study. Data sharing is not applicable to this article.

## References

[1] Humbert M., Kovacs G., Hoeper M.M., Badagliacca R., Berger R.M.F., Brida M., Carlsen J., Coats A.J.S., Escribano-Subias P., Ferrari P. (2022). 2022 ESC/ERS Guidelines for the diagnosis and treatment of pulmonary hypertension. Eur. Heart J..

[2] Ivy D., Rosenzweig E.B., Abman S.H., Beghetti M., Bonnet D., Douwes J.M., Manes A., Berger R.M.F. (2024). Embracing the challenges of neonatal and paediatric pulmonary hypertension. Eur. Respir. J..

[3] Rosenzweig E.B., Abman S.H., Adatia I., Beghetti M., Bonnet D., Haworth S., Ivy D.D., Berger R.M.F. (2019). Paediatric pulmonary arterial hypertension: Updates on definition, classification, diagnostics and management. Eur. Respir. J..

[4] Miles K.G., Dorobantu D.M., Douwes J.M., Frank B.S., Jone P.-N., Muthurangu V., Ivy D.D., Nawaytou H., Friedberg M.K. (2026). Current state of RV multimodality imaging in pediatric pulmonary hypertension: Current evidence, knowledge gaps, and future research directions. Circ. Cardiovasc. Imaging.

[5] Vonk Noordegraaf A., Chin K.M., Haddad F., Hassoun P.M., Hemnes A.R., Hopkins S.R., Kawut S.M., Langleben D., Lumens J., Naeije R. (2019). Pathophysiology of the right ventricle and of the pulmonary circulation in pulmonary hypertension: An update. Eur. Respir. J..

[6] Naeije R., Richter M.J., Rubin L.J. (2022). The physiological basis of pulmonary arterial hypertension. Eur. Respir. J..

[7] Jone P.N., Schafer M., Pan Z., Ivy D.D. (2019). Right ventricular-arterial coupling ratio derived from 3-dimensional echocardiography predicts outcomes in pediatric pulmonary hypertension. Circ. Cardiovasc. Imaging.

[8] Dufva M.J., Ivy D., Campbell K., Lam A., Rauff A., Breeman K.T.N., Douwes J.M., Berger R.M.F., Kheyfets V.O., Hunter K. (2021). Ventricular-vascular coupling is predictive of adverse clinical outcome in paediatric pulmonary arterial hypertension. Open Heart.

[9] Simpkin C.T., Ivy D.D., Friedberg M.K., Burkett D.A. (2024). Use of right ventricular free-wall strain in a multivariable estimate of right ventricular-arterial coupling in pediatric pulmonary arterial hypertension. Circ. Cardiovasc. Imaging.

[10] Miles K.G., Ta H.T., Bjorkman K.R., Gao Z., Ollberding N.J., Hirsch R., Ivy D.D., Lang S.M., Cash M., Magness M. (2025). Right atrial function is impaired in pediatric pulmonary arterial hypertension: A multi-center cardiovascular magnetic resonance study. J. Cardiovasc. Magn. Reson..

[11] Jone P.N., Schafer M., Li L., Craft M., Ivy D.D., Kutty S. (2017). Right atrial deformation in predicting outcomes in pediatric pulmonary hypertension. Circ. Cardiovasc. Imaging.

[12] Frank B.S., Schafer M., Thomas T.M., Ivy D.D., Jone P.N. (2021). Longitudinal assessment of right atrial conduit fraction provides additional insight to predict adverse events in pediatric pulmonary hypertension. Int. J. Cardiol..

[13] Kumar S., Vadlamudi K., Kaddoura T., Bobhate P., Goot B.H., Elgendi M., Jain S., Colen T., Khoo N.S., Adatia I. (2018). Active right atrial emptying fraction predicts reduced survival and increased adverse events in childhood pulmonary arterial hypertension. Int. J. Cardiol..

[14] Jone P.N., Ivy D.D., Hauck A., Karamlou T., Truong U., Coleman R.D., Sandoval J.P., del Cerro Marín M.J., Eghtesady P., Tillman K. (2023). Pulmonary hypertension in congenital heart disease: A scientific statement from the American Heart Association. Circ. Heart Fail..

[15] Abman S.H., Hansmann G., Archer S.L., Ivy D.D., Adatia I., Chung W.K., Hanna B.D., Rosenzweig E.B., Raj J.U., Cornfield D. (2015). Pediatric pulmonary hypertension: Guidelines from the American Heart Association and American Thoracic Society. Circulation.

[16] Berger R.M.F., Beghetti M., Humpl T., Raskob G.E., Ivy D.D., Jing Z.-C., Bonnet D., Schulze-Neick I., Barst R.J. (2012). Clinical features of paediatric pulmonary hypertension: A registry study. Lancet.

[17] Aly D., Ramlogan S., France R., Schmidt S., Hinzman J., Sherman A., Goudar S.P., Forsha D. (2021). Intervendor Agreement for Right Ventricular Global Longitudinal Strain in Children. J. Am. Soc. Echocardiogr..

[18] van der Ven J.P.G., Sadighy Z., Valsangiacomo Buechel E.R., Sarikouch S., Robbers-Visser D., Kellenberger C.J., Kaiser T., Beerbaum P., Boersma E., Helbing W.A. (2020). Multicentre reference values for cardiac magnetic resonance imaging derived ventricular size and function for children aged 0-18 years. Eur. Heart J. Cardiovasc. Imaging..

[19] Beghetti M., Schulze-Neick I., Berger R.M.F., Ivy D.D., Bonnet D., Weintraub R.G., Saji T., Yung D., Mallory G.B., Geiger R. (2016). Haemodynamic characterisation and heart catheterisation complications in children with pulmonary hypertension: Insights from the Global TOPP Registry. Int. J. Cardiol..

[20] Apitz C., Hansmann G., Schranz D. (2016). Hemodynamic assessment and acute pulmonary vasoreactivity testing in the evaluation of children with pulmonary vascular disease. Heart.

[21] Douwes J.M., Humpl T., Bonnet D., Beghetti M., Ivy D.D., Berger R.M.F., Weintraub R.G., Geiger R., Marx M., Jing Z.-C. (2016). Acute vasodilator response in pediatric pulmonary arterial hypertension: Current clinical practice from the TOPP Registry. J. Am. Coll. Cardiol..

[22] Koestenberger M., Friedberg M.K., Nestaas E., Michel-Behnke I., Hansmann G. (2016). Transthoracic echocardiography in the evaluation of pediatric pulmonary hypertension and ventricular dysfunction. Pulm. Circ..

[23] Koestenberger M., Apitz C., Abdul-Khaliq H., Hansmann G. (2016). Transthoracic echocardiography for the evaluation of children and adolescents with suspected or confirmed pulmonary hypertension. Heart.

[24] Okumura K., Humpl T., Dragulescu A., Mertens L., Friedberg M.K. (2014). Longitudinal assessment of right ventricular myocardial strain in relation to transplant-free survival in children with idiopathic pulmonary hypertension. J. Am. Soc. Echocardiogr..

[25] Lammers A.E., Marek J., Diller G.P., Haworth S.G., Moledina S. (2023). Prognostic value of transthoracic echocardiography in children with pulmonary arterial hypertension. J. Am. Heart Assoc..

[26] Ta H.T., Critser P.J., Schafer M., Ollberding N.J., Taylor M.D., Di Maria M.V., Hirsch R., Ivy D.D., Frank B.S. (2023). Ventricular global function index is associated with clinical outcomes in pediatric pulmonary hypertension. J. Cardiovasc. Magn. Reson..

[27] Said F., Haarman M.G., Roofthooft M.T.R., Hillege H.L., Ploegstra M.-J., Berger R.M.F. (2020). Serial measurements of N-terminal pro-B-type natriuretic peptide serum level for monitoring pulmonary arterial hypertension in children. J. Pediatr..

[28] Zijlstra W.M.H., Ploegstra M.J., Vissia-Kazemier T., Roofthooft M.T.R., du Marchie Sarvaas G., Bartelds B., Rackowitz A., van den Heuvel F., Hillege H.L., Plasqui G. (2017). Physical activity in pediatric pulmonary arterial hypertension measured by accelerometry: A candidate clinical endpoint. Am. J. Respir. Crit. Care Med..

[29] Ploegstra M.J., Ferreira R.J., Lokhorst C., Gouwy E., Schwartz S.S., Haarman M.G., Villeneuve M., Stamatiadis D., Douwes J.M., Berger R.M.F. (2025). Physical activity measured by hip-anchored accelerometry in pediatric pulmonary hypertension: Association with disease severity and estimation of minimal important differences. Chest.

[30] Latus H., Meierhofer C. (2021). Role of cardiovascular magnetic resonance in pediatric pulmonary hypertension—Novel concepts and imaging biomarkers. Cardiovasc. Diagn. Ther..

[31] Kellenberger C.J. (2025). Magnetic resonance imaging of pulmonary hypertension. Pediatr. Radiol..

[32] Chu H., Ferreira R.J., Lokhorst C., Douwes J.M., Haarman M.G., Willems T.P., Berger R.M.F., Ploegstra M.-J. (2025). Predicting pulmonary hemodynamics in pediatric pulmonary arterial hypertension using cardiac magnetic resonance imaging and machine learning: An exploratory pilot study. Int. J. Cardiovasc. Imaging..

[33] Simpkin C.T., Frank B.S., Liu Y., Pan Z., Ivy D.D., Burkett D.A. (2025). Echocardiographic prediction of hemodynamics during acute vasoreactivity testing in pediatric pulmonary arterial hypertension. Echocardiography.

[34] Ploegstra M.J., Ivy D.D., Beghetti M., Bonnet D., Alehan D., Ablonczy L., Mattos S., Bowers D., Humpl T., Berger R.M.F. (2024). Long-term outcome of children with newly diagnosed pulmonary arterial hypertension: Results from the global TOPP registry. Eur. Heart J. Qual. Care Clin. Outcomes.

[35] Sahay S., Visovatti S., Tonelli A.R., Villasmil Hernandez N., Austin E.D., Badagliacca R., Berger R.M.F., Boucly A., Chen Y., Church C. (2025). International Society for Heart and Lung Transplantation consensus statement on risk stratification in pulmonary arterial hypertension. J. Heart Lung Transplant..

[36] Hjalmarsson C., Thakur T., Rådegran G., Björklund E., Wåhlander H., Nisell M., Papageorgiou J., Söderberg S., Lautsch D., Kjellström B. (2025). Risk assessment models and event-free survival in pulmonary arterial hypertension. Pulm. Circ..

